# Expression and Purification of P43 *Toxoplasma gondii* Surface Antigen

**Published:** 2012

**Authors:** K Khanaliha, MH Motazedian, B Sarkari, M Bandehpour, Z Sharifnia, B Kazemi

**Affiliations:** 1 Department of Parasitology and Mycology, School of Medicine, Shiraz University of Medical Sciences, Shiraz, Iran; 2 Cellular and Molecular Biology Research Center, Shahid Beheshti University of Medical Sciences, Tehran, Iran; 3 Biotechnology Department, Shahid Beheshti University of Medical Sciences, Tehran, Iran

**Keywords:** *Toxoplasma*, Tachyzoite, Bradyzoite, Recombinant P43

## Abstract

**Background:**

*Toxoplasma gondii* is an obligate intracellular protozoan parasite, capable of infecting all species of mammals including man. Congenital toxoplasmosis is more important during pregnancy for the first time. In this study we expressed and purified *P43 Toxoplasma gondii* tachyzoite and bradyzoite specific surface antigen.

**Methods:**

The recombinant pGEMEX-1 contained *Toxoplasma* P43 coding sequence was transformed into *E. coli* and mass cultured in LB medium contained 100 μg/ml ampicillin at 37°C over night. The T7 promoter was induced by 1mM isopropyl-1-thio-ß-D-galactopyranoside (IPTG. Recombinant protein was purified by affinity chromatography and confirmed by gel diffusion dot blot and western blot,-using specific anti *Toxoplasma* antibodies.

**Results:**

Recombinant plasmid was induced by IPTG and analyzed by SDS-PAGE. Recombinant protein was confirmed by Western-blot and dot blot using anti human *Toxoplasma* antibody.

**Conclusion:**

Recombinant *Toxoplasma* P43 was produced successfully.

## Introduction

*Toxoplasma gondii* is an obligate intracellular protozoan parasite that causes one of the most common parasitic infections in the world and affecting a wide range of hosts including human, domestic animals and birds (1). Toxoplasmosis is generally asymptomatic in immuonocompetent people whereas intrauterine transmission of the parasite during pregnancy can result in neonatal complications. Congenital toxoplasmosis is more important when the mother acquire the infection during pregnancy for the first time (2). Toxoplasmosis in patients who are severely immunocompromised causes encephalitis.

Recently main attention has been attracting toward the surface molecules of the parasite (3, 4). The surfaces of tachyzoite and bradyzoite are covered with antigens named as SAG (surface antigen), which is linked to GPI (glycosyle phosphatydyl inositol) (5–7). Some of these specific molecules including P30 (SAG1) and P22 (SAG2) are exclusively in the surface of tachyzoite whereas others such as P43(SAG3) occurs on the surface of both tachyzoite and bradyzoite (8–10).

It has been demonstrated that SAG3 but not SAG1 interacts specifically with cellular HSPGs (heparin sulfate proteoglycans) and specific interaction of SAG3 with cellular proteoglycans mediates the adsorption of *T. gondii* to host cells (11). The mutant forms of tachyzoites lacking SAG3, another surface GPI-anchored protein, showed less virulence in vivo than wild-type, indicating that SAG3 could also be involved in the infection process of *T. gondii* (12).

*Toxoplasma* P43 have been cloned and sequenced for the first time by Cesbron-Delauw and colleagues in 1994 (13) and followed by Fux and colleagues in 2003. Furthermore this protein (antigen) has been cloned and sequenced by Kazemi et al. (4).

In this study we expressed and purified a 1158 bp fragment of *T. gondii* coding P43, to evaluate its efficacy in detecting of *T. gondii* antibodies.

## Materials and Methods

### P43 gene

Recombinant pGEMEX-1 expression vector containing P43 gene (4) was confirmed by PCR, using specific primers and restriction analysis by PstI. There is no restriction site for PstI on pGEMEXI plasmid but there is one restriction site for PstI at position 748-753 on P43 gene.

PCR product was electrophoresed on 1.5% agarose gel and visualized by UV transilluminator.

### Protein expression

The recombinant pGEMEX-1 plasmid was transformed into *Escherichia coli* BL21 *(DE3)* and JM109 competent cells. A single colony was inoculated in LB medium (Merck, Germany) containing 100 μg/ml ampicillin and incubated overnight at 37°C. Cells was subcultured, using 10-fold fresh LB medium containing 100µg/ml ampicillin and shacked at 200 rpm in 37°C until the OD_600_ = 0.7. The plasmid promoter was induced by 1 mM isopropyl β- D-1-thiogalactopyranoside (IPTG) for 5 hours****. Sampling was done before and after induction in one hour intervals. The bacterial cells were harvested by centrifugation at 8000 rpm for 15 minutes. The expressed protein was subjected to SDS-PAGE and western blot analysis.

### Protein purification

Recombinant protein was purified, using The T7•Tag Affinity Purification Kit (Novagen, Madison Wisconsin, USA) according to the manufacturer's instruction with some modifications. The cell pellet was extracted from 25 ml of liquid culture and resuspended in 5 ml of equilibration buffer with final concentration of 1 mM PMSF (Sigma, St****. Louis****, Missouri, USA) on ice for 2 hours. The suspension was then sonicated and centrifuged (10000 rpm for 15 min at 4°C). The supernatant was transferred to column containing 2 ml of equilibrated resin. The column was incubated overnight at 4°C and washed with 10 ml of washing buffer (KH2PO4, Na2HPO4, KCl, NaCl, and Tween 20). Finally elution buffer was added (1mM citric acid) and incubated for 2 hours and recombinant P43 protein was eluted and neutralized with 1mM Tris-HCl. The protein concentration was measured by biophotometer (Eppendorf, Hamburg, Germany).

### SDS- PAGE and western blot analysis

Recombinant proteins was subjected to 12% gradient SDS-PAGE with the molecular protein marker (Fermentas, Lithuania cat) and western blot analysis using specific anti *Toxoplasma* antibody of patients sera was performed as previously described (14).

## Results

**Fig. 1:**
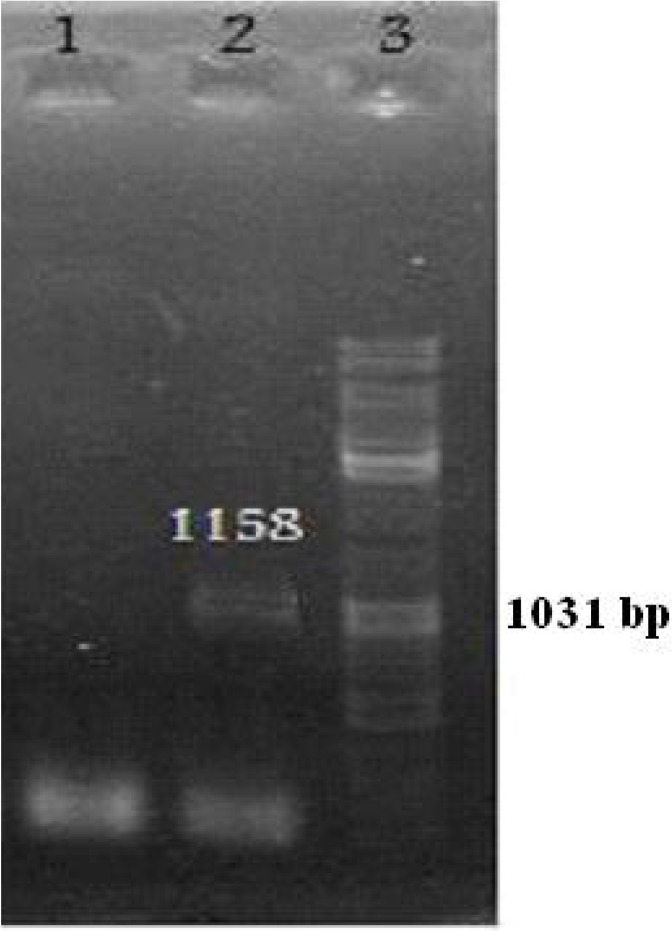
Confirmation of recombinant pGEME43.A PCR was performed using Toxo43 primer and PCR product was separated by electrophoresis on a 1.5% agarose gel. Lane 1 pGEMEX-1 without the SAG3 insert:. Lane 2: pGEMEX-1 containing the SAG3 insert (1158bp). Lane 3: 100bp DNA ladder marker

**Fig. 2:**
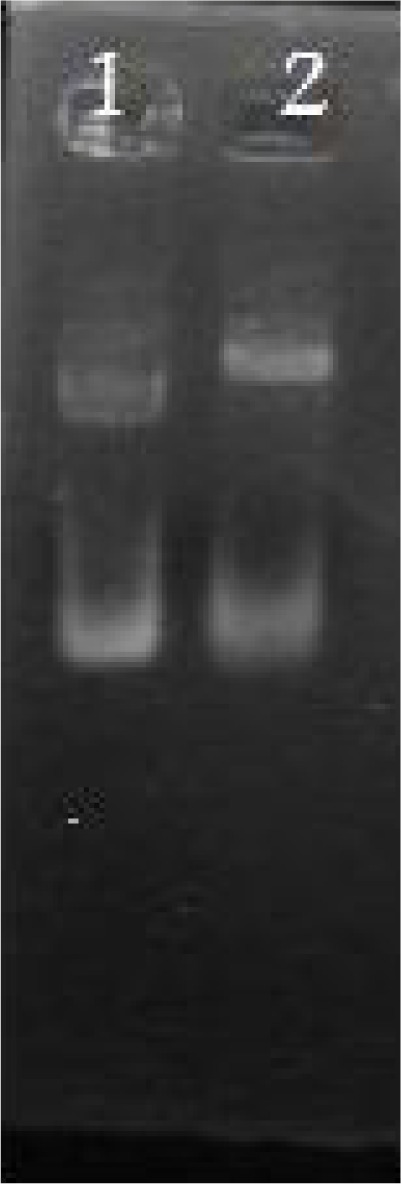
Restriction analysis of the pGEMEX43. Lane 1: un digested pGEMEX-1. Lane2: recombinant pGEMEX43 digested by PstI. There is no restriction site for PstI on pGEMEXI plasmid but there is one restriction site for PstI at position 748-753 on P43 gene

**Fig. 3:**
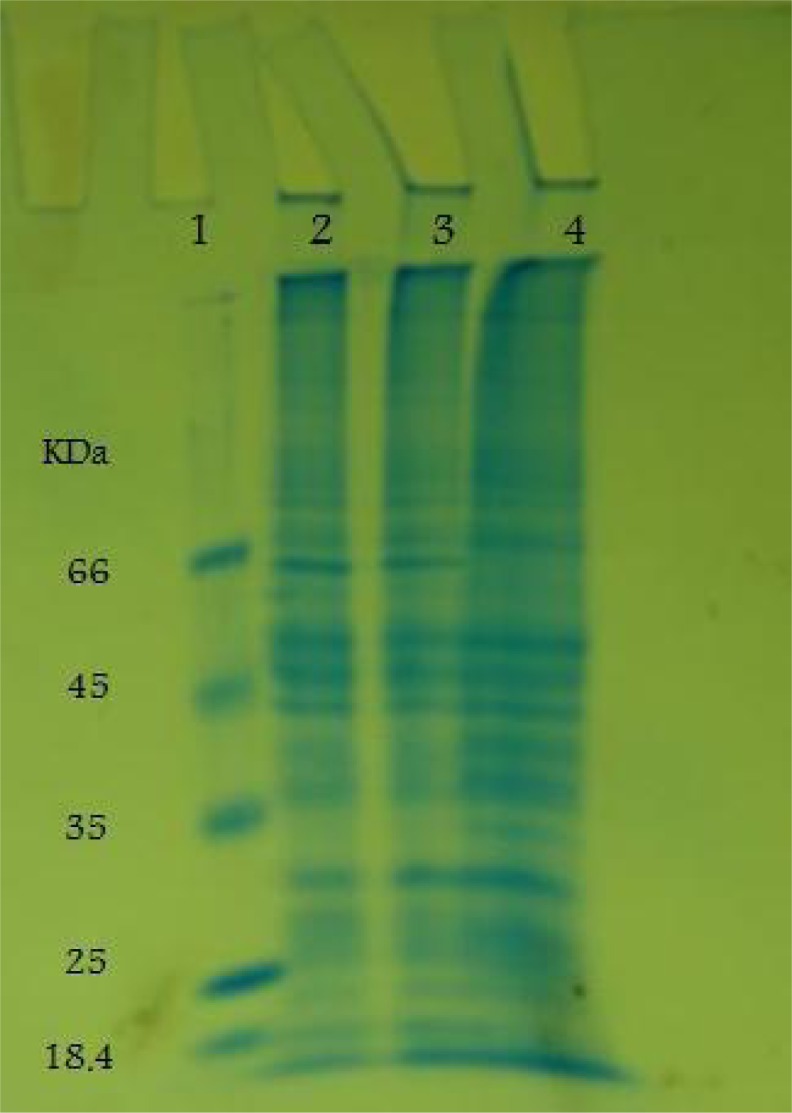
SDS-PAGE analysis of rSAG3 expression using 12% SDS-PAGE. lane 1: protein marker. Lane 2: 65 kDa expressed SAG3 with fusion protein 3 hours after induction. Lane 3: expressed of SAG3 fusion protein 5hours after induction. Lane 4: lysate of non-induced bacterial cells

Recombinant pGEME43 was confirmed by PCR (Fig. 1) and restriction digestion with PstI restriction enzyme (Fig. 2). Recombinant plasmid was mass cultured and induced by IPTG and protein was electrophoresed on SDS-PAGE. Molecular weight of fusion protein is 74 kDa (43kDa for SAG3 and 31 kDa for T7gene 10 protein) (18) but a 65 kDa of expressed protein was detected 3 and 5 hours after induction by IPTG (Fig. 3).

**Fig. 4:**
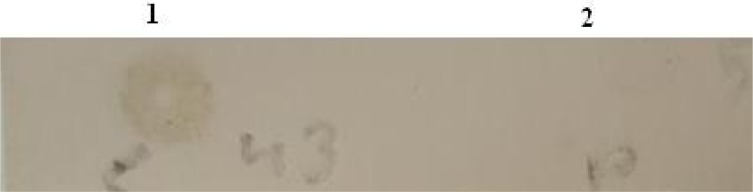
Dot blot analysis of rSAG3 protein reacted with *Toxoplasma gondii*- positive serum. Left: recombinant SAG3. Right: lysate of bacterial cells from non-induced culture

**Fig. 5:**
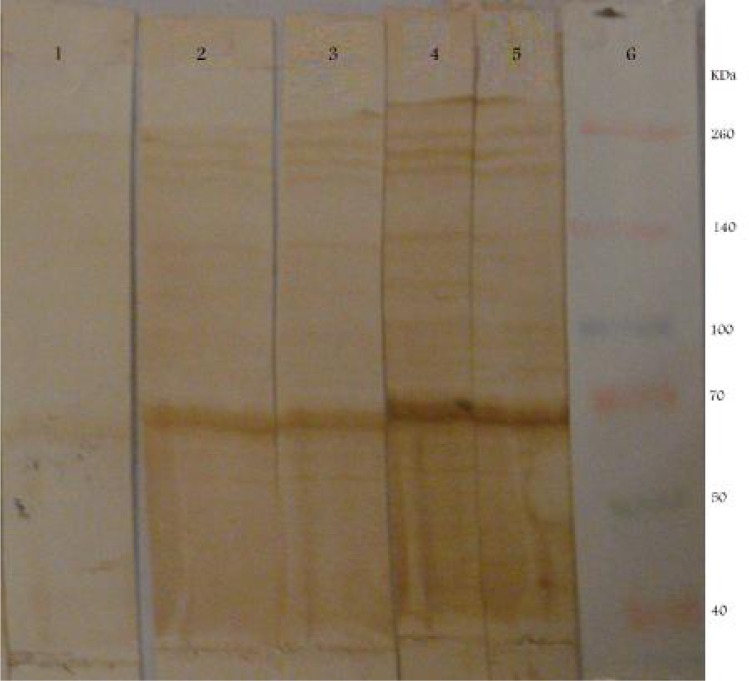
Western blot analysis of the rSAG3 protein using *Toxoplasma gondii*- positive serum. Lane 1: lysate of non-induced bacterial cells. Lane2: expresed SAG3 2 hours after induction. Lane3: expressed SAG3 3 hours after induction. Lane4: expressed SAG3 5 hours after induction. Lane 5: expressed SAG3 7 hours after induction. Lane 6: protein marker

**Fig. 6:**
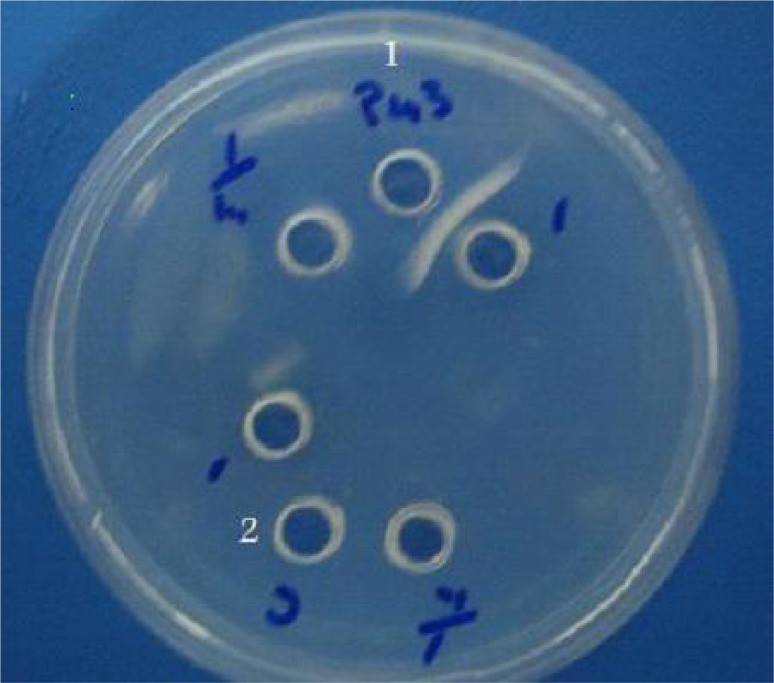
Gel difiussion of the rSAG3 using Toxoplasma gondii-positive serum. Well 1: recombinant SAG3. Well 2: lysate of bacterial cells from non-induced culture

The reactivities of the recombinant proteins were determined using human sera by dot blot and western blot analysis (Figs. 4 and 5). Figure 6 shows reaction of recombinant SAG3 and human IgG pooled serum in gel diffusion in comparison with bacterial cells without plasmid.

## Discussion

*Toxoplasma gondii* is an obligate intracellular protozoan parasite and affecting a wide range of hosts including human (1). Toxoplasmosis is generally asymptomatic whereas intrauterine transmission of the parasite during pregnancy can result in neonatal complications. Diagnosis is based on serological methods and specific antigens are a critical. There are some specific surface molecules on *Toxoplasma* tachyzoite and bradyzoite like SAG3. Recombination is an easy method for accessing to this specific protein.

Recently, recombinant proteins have been used in an attempt to improve the serological diagnosis of toxoplasmosis (15–17). In this study recombinant pGEMEX-1 containing cds of *Toxoplasma* SAG3 (4) was expressed and purified using T7 tag affinity chromatography column according to the manufacturers protocol and analyzed by dot and western blot using human infected *Toxoplasma* serum as primary antibody.

In the current study we expressed and purified recombinant SAG3 in *E. coli* expression system for using in the diagnosis of human toxoplasmosis by ELISA system.
